# A system for reporting and evaluating adverse drug reactions of herbal medicine in Taiwan from 1998 to 2016

**DOI:** 10.1038/s41598-021-00704-w

**Published:** 2021-11-02

**Authors:** Hen-Hong Chang, Su-Yin Chiang, Pei-Chun Chen, Chia-Han Tsai, Rong-Chi Yang, Chiu-Lin Tsai, Tsung-hsiu Wu, Yow-Wen Hsieh, Yu-Chun Lin, Yung-Te Kuo, Kuan-Chung Chen, Hsueh-Ting Chu

**Affiliations:** 1 Graduate Institute of Integrated Medicine, College of Chinese Medicine, and Chinese Medicine Research Center, China Medical University, No. 91, Hsueh-Shih Road, North District, Taichung, 40402 Taiwan, ROC; 2 Department of Chinese Medicine, China Medical University Hospital, Taichung, Taiwan; 3 School of Chinese Medicine, College of Chinese Medicine, China Medical University, Taichung, Taiwan; 4 Department of Public Health, College of Public Health, China Medical University, Taichung, Taiwan; 5 Division of Chinese Internal Medicine, Department of Chinese Medicine, China Medical University Hospital, Taichung, Taiwan; 6 School of Traditional Chinese Medicine, Chang Gung University, Taoyuan, Taiwan; 7 Traditional Chinese Medicine Pharmacy, China Medical University Hospital, Taichung, Taiwan; 8 Traditional Chinese Medicine Pharmacy, Taipei City Hospital, Taipei, Taiwan; 9 Department of Pharmacy, China Medical University Hospital, Taichung, Taiwan; 10 School of Pharmacy, College of Pharmacy, China Medical University, Taichung, Taiwan; 11 Department of Traditional Chinese Medicine, Chinese Medicine Department, China Medicine University Hospital, Taichung, Taiwan; 12 Traditional Chinese Medicine Pharmacy, Chang Gung Medical Foundation, Linkou Chang Gung Memorial Hospital, Taoyuan, Taiwan; 13 Department of Computer Science and Information Engineering, College of Computer Science, Asia University, No. 500, Lioufeng Road, Wufeng, Taichung, 41354 Taiwan, ROC

**Keywords:** Databases, Drug safety

## Abstract

The Taiwan Adverse Drug Reaction Reporting System for Herbal Medicine (TADRRS-HM) has systematically documented suspected adverse events from adverse drug reaction (ADR) reports from 1998 (prior to its formal establishment in 2001) and evaluates safety profiles of herbal medicines. This article describes findings from 2079 ADR reports filed between 1998 and 2016: 941 reports involved single herbs and 87 involved folk herbals; 842 were generated from clinical trials, while 209 ADR reports involving foods, health foods, dietary supplement foods and herbal cuisine were grouped as Other. Severity assessments using the Modified Hartwig and Siegel scale classified 72.4% of ADRs as mild, 17.4% as moderate and 6.5% as severe. System Organ Class classification of the ADRs identified gastrointestinal system disorders as the most common (33.4%), followed by skin and subcutaneous tissue disorders (21.2%). The TADRRS-HM records indicate that herbal medicines may cause a wide range of ADRs. Aconiti Radix, Xiao-Qing-Long-Tang, and *Datura suaveolens* were the most commonly reported single herb, herbal formula, and folk herbal, respectively. The data indicate that herbal medicines may cause a wide range of ADRs. This system will confer long-term benefits for the development of Taiwan’s herbal medicines adverse reaction database and facilitate epidemiological analysis.

## Introduction

Herbal medicine is a distinct medical system that has evolved over thousands of years and is accessible in over 170 countries worldwide^1^. The growing popularity of herbal medicines has prompted increased attention concerning their safety, quality, and appropriate use^2–4^. The common assumption that herbal medicines are bereft of any major side effects has fostered rampant over-the-counter use, but such compounds can result in bodily harm due to their complex chemical compositions and often uncertain pharmacology and toxicology^5^. Thus, the Department of Health in Taiwan has emphasized the importance of monitoring adverse drug reactions (ADRs) associated with herbal medicines, including appropriate and safe intake^6^. Currently, most reports of adverse reactions to herbal medicines have emanated from China or other countries^7–11^, as Taiwan lacks a comprehensive ADR database for herbal medicines.

In 1982, Taiwan initiated a program called Good Manufacturing Practice (GMP)^12^ and then on February 5, 1993, the government announced the Pharmaceutical Affairs Law. These laws and regulations have enforced strict requirements with respect to the quality of drug production and management. Moreover, the effects of any new drug must be fully investigated prior to its market entry, and the drug must undergo follow-up safety evaluations after market entry. Since July 7, 1993, all new drugs in Taiwan are subject to postmarketing surveillance for a period of 7 years. One of the most important links in this process is a system for reporting adverse reactions of herbal medication.

With the widespread clinical use of herbal medication, the frequency of adverse reactions has increased^13,14^. The active ingredients in any given herbal medicines are complex; this complexity is enhanced when several herbal medicines are concomitantly used in a formula. Documentation and reporting of adverse reactions is a key imperative for developing an evidence base that evaluates the safety of herbal medicines. Taiwan’s reporting system for ADRs is largely programmed for Western medicine. However, the principles and format for evaluation of Western medicine are not suitable for evaluation of herbal medicines. Therefore, during the 1990s, the Committee on Chinese Medicine and Pharmacy, Department of Health, Executive Yuan, entrusted a project for developing an adverse drug reporting system to the Center for Traditional Chinese Medicine (TCM) in Chang Gung Memorial Hospital. This reporting system is tailored to the unique characteristics of herbal medicine. It also compiles the expert opinions of TCM physicians, pharmacologists, epidemiologists, and toxicologists. The Taiwan Adverse Drug Reaction Reporting System for Herbal Medicine (TADRRS-HM) was officially established in 2001, 3 years after data collection was initiated. Training has been provided at the local centers; in addition, a regularly updated website has been established for dissemination of information. The TADRRS-HM system aims to inform an expert group on adverse reactions of herbal medicine while creating a valuable database. Periodic meetings are held to encourage reporting from local centers and to keep them up-to-date with new developments. For ADR events, three types of reporting systems are available: the spontaneous reporting system, prescription event monitoring^15^; and epidemiology and clinical trial methods. The majority of cases in the TADRRS-HM are generated by spontaneous reporting, which is the most common method used to report ADRs in many countries^16–19^.

Taiwan has witnessed remarkable improvements in the standard of medical treatment; the appropriate use of drugs is already considered to be an essential element of safe medical practice. Establishment of a system for documentation and investigation of the ADRs of herbal medicines was a further step in this direction; another aim was to evaluate the quality of these medicines and the technology used for their production. This system reinforces the concept of re-evaluation of a drug after its entry into the market (i.e., the equivalent of a phase IV clinical study).

The TADRRS-HM is a nationwide database. In this study, our analysis of ADR records from this database for the period of 1998 through 2016 documented all adverse events (AEs) associated with herbal medicines to evaluate the characteristics of AEs and to determine the causality and severity of ADRs, as well as the most frequently reported herbals and formulas associated with those ADRs. We expect that our findings will help to prevent adverse reactions associated with the use of herbal medicines.

## Methods

### Establishment of the Taiwan adverse drug reaction reporting system for herbal medicine

The scope of ADRs to herbal medicine encompasses adverse reactions caused by traditional herbal medicines and local herbals used in folk medicine. The main characteristics of the TADRRS-HM are summarized below:

1. Recording of all adverse reactions of herbal medicine (including those resulting from over-the-counter use).
2. Establishment of a set of principles for the reporting system, including standardized formats for reports and investigations.
3. Establishment of a step-by-step process to facilitate expert medical treatment of each case. The standardized formats are verified by an assistant, and each ADR event is evaluated and disaggregated by pharmacists and doctors. This process also involves a group of professional medical experts.
4. Compilation of all adverse reaction cases, analyses, and evaluations.
5. Organization of workshops to train medical workers on the reporting system.
6. Creation of an “Adverse Drug Reaction Reporting System for Herbal Medicine in Taiwan” website.

Five local reporting branches and five main reporting hospitals were set up. Their main activities include: 1) receiving case reports from both the medical community and the public; 2) follow-up and evaluation of these cases; 3) establishment of a step-by-step investigative process for each case; 4) creation of a consulting group comprising of medical professionals; 5) creation of an adverse reaction database; 6) creation of a regularly updated website to post relevant and timely information; and 7) organizing workshops at reporting centers to train and encourage medical workers to report cases of adverse reactions.

Each reporter is required to report his/her name, telephone number, name of the workplace, and personal address. The case serial number is recorded for follow-up verification. The Department of Health and Welfare in Taiwan and the Taiwan TCM Adverse Reaction Reporting Center are responsible for protecting the rights and confidentiality of the case and the person reporting the adverse reactions. In the case of ADRs arising during clinical trials conducted in hospitals, the drug companies and industries involved with the medicine in question are permitted access to the personal information of the case, unless the person who is reporting requests otherwise. When publicly releasing personal information, these institutions must comply with clearly defined legal restrictions. Moreover, data from these case reports are not allowed to be used for legal purposes. Folk herbals are defined as herbal medicines not included in the Taiwan Herbal Pharmacopoeia^20,21^ and Pharmacopoeia of the People’s Republic of China (Chinese Pharmacopoeia) Volume 1^22^.

### Definition of an adverse reaction to herbal medicine

An ADR is defined as any appreciably harmful or unpleasant reaction associated with an intervention relating to the use of a medicinal product; ADRs include reactions occurring due to error, misuse or abuse, and reactions to medicines that are unlicensed or being used off-label in addition to the authorized use of a medicinal product in normal doses^23^. The International Conference on Harmonization (ICH) defines a “serious” ADR as any untoward medical occurrence that at any dose: (i) results in death; (ii) is life-threatening; (iii) requires patient hospitalization or prolongs the existing hospitalization; (iv) results in persistent or significant disability/incapacity; or (v) a congenital anomaly/birth defect.

### Causality analysis

ADR causality was determined by the Naranjo algorithm^24^, with Naranjo scores analyzing the probability of ADRs caused by single herbs, herbal formulas, or folk herbals scored as “doubtful”, “possible”, or “definite”^24^, whereby a total score of ≤ 0 was interpreted as “doubtful”: the reaction was likely related to factors other than a drug; a total score of 1–4 was interpreted as “possible”: the reaction (1) followed a temporal sequence after a drug, (2) possibly followed a recognized pattern to the suspected drug, and (3) could be explained by characteristics of the patient’s disease; a total score of 5–8 was interpreted as “probable”: the reaction (1) followed a reasonable temporal sequence after a drug, (2) followed a recognized response to the suspected drug, (3) was confirmed by withdrawal but not by exposure to the drug, and (4) could not be reasonably explained by the known characteristics of the patient’s clinical state; and a total score of ≥ 9 was interpreted as definite: the reaction (1) followed a reasonable temporal sequence after a drug or in which a toxic drug level had been established in body fluids or tissues, (2) followed a recognized response to the suspected drug, and (3) was confirmed by improvement on withdrawing the drug and reappeared on re-exposure. Severity of the clinical manifestations of AEs was assessed as mild, moderate, or severe by the Modified Hartwig and Siegel scale^25^, while ADRs were classified by System Organ Class (SOC).

We used Common Terminology Criteria for Adverse Events (CTCAE) 4.0 terms to code the narrative symptoms of each ADR. SOCs of CTCAE terms are grouped by MedDRA. According to CTCAE 4.0, each term satisfies the lowest level term (LLT) of MedDRA, which can be used to describe in greater detail the ADR information in the TADRRS-HM database. We therefore used only the LLT instead of MedDRA high-level terms (HLT) or preferred terms (PT).

### Statistical analysis

The means, percentages and frequencies are reported for all 1028 cases involving herbal medicine in this analysis. We analyzed data on patient gender and age, as well as the causality and severity of ADRs.

## Results

### Basic information of the ADR reports

A total of 2079 ADR reports were recorded during the 19-year study period (Table 1). A total of 941 reports involved single herbs, 87 involved folk herbals, 842 involved herbal medicines used in clinical trials, and 209 ADR reports involved foods, health foods, dietary supplement foods and herbal cuisine.

**Table 1:** ADR reports analyzed from the TADRRS-HM in Taiwan (1998–2016).

	1998–2002	2003–2007	2008–2012	2013–2016	Total
	n (%)	n (%)	n (%)	n (%)	n (%)
Herbal medicine*	60 (32.4)	339 (41.3)	406 (52.4)	136 (45.5)	941 (45.3)
Folk herbals**	9 ( 4.9)	40 ( 4.9)	22 ( 2.8)	16 ( 5.3)	87 ( 4.2)
RCT	70 (37.8)	337 (41.1)	319 (41.2)	116 (38.8)	842 (40.5)
Other^#^	46 (24.9)	104 (12.7)	28 (3.6)	31 (10.4)	209 (10.1)
Total	185 (100)	820 (100)	775 (100)	299 (100)	2079 (100)

*TADRRS-HM* Taiwan Adverse Drug Reaction Reporting System for Herbal Medicine.

*Herbal medicines included in the Taiwan Herbal Pharmacopeia or Chinese Pharmacopoeia Volume 1, as well as single herbs and herbal formulas registered as medicinal products, used in real-world conditions.

**Herbals not included in the Taiwan Herbal Pharmacopeia or Chinese Pharmacopoeia Volume 1.

^#^Involving herbals from clinical trials, foods, health foods, dietary supplement foods and Chinese herbal cuisine.

**Figure 1:**
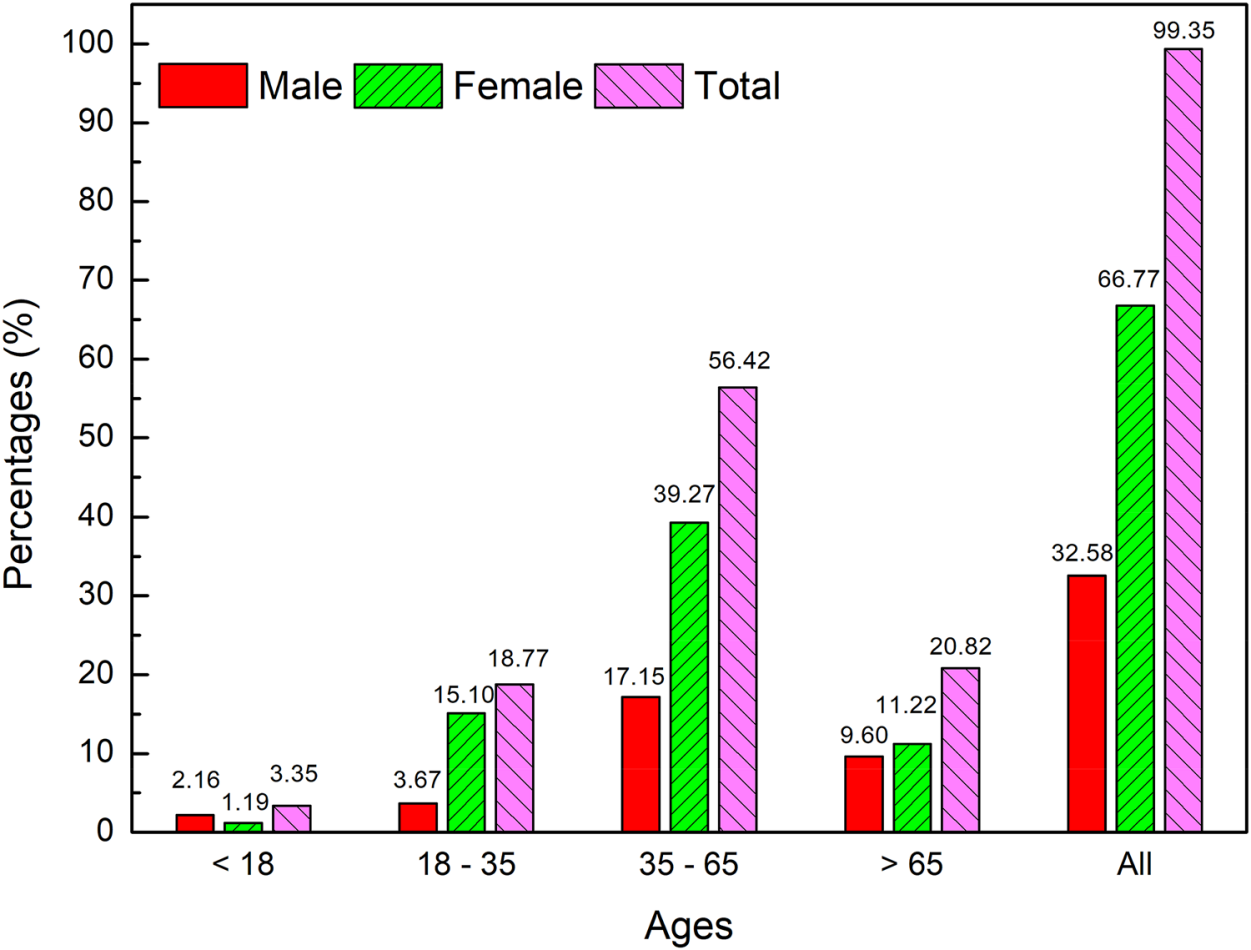
Distributions of age and sex in the 1028 ADR reports involving single herbs, herbal formulae and folk herbals analyzed from the TADRRS-HM (1998–2016).

The age and sex distribution of cases in the 1028 ADRs involving single herbs and folk herbals are presented in Fig. 1, which shows that most ADRs involved adults aged 36–65 years and the majority of cases were female.

### Origins of 1028 ADR reports

As shown in Table 2, most of the 1028 ADR reports involving single herbs and folk herbals were submitted by medical personnel (n = 990; 96.3%), the majority of whom were pharmacists (n = 621; 60.4%); far fewer ADR reports were submitted by doctors (n = 267; 26.0%) and almost none by nurses (n = 3; 0.3%). Taiwan is divided into four Regions (Northern, Central, Southern and Eastern). The Northern Region contains the capital Taipei, where most people live and the majority of medical centers are located. Up until the end of 2016, the majority of the ADR reports analyzed in this study originate from the Northern Region.

**Table 2:** 1028 ADR reports involving herbal medicines in the TADRRS-HM (1998–2016).

	Total numbers of ADR reports				
	1998–2002	2003–2007	2008–2012	2013–2016	Total
	n (%)	n (%)	n (%)	n (%)	n (%)
**Types of reporters**					
Medical personnel	59 (85.5)	360 (95)	426 (99.5)	145 (95.4)	990 (96.3)
Doctors	19 (27.5)	121 (31.9)	100 (23.4)	27 (17.8)	267 (26.0)
Pharmacists	40 (58.0)	212 (55.9)	279 (65.2)	90 (59.2)	621 (60.4)
Nursing staff	0 (0)	2 (0.5)	1 (0.2)	0 (0)	3 (0.3)
Others^#^	0 (0)	25 (6.6)	46 (10.7)	28 (19.3)	99 (10.0)
Researchers	5 (7.3)	11 (2.9)	0 (0)	0 (0)	16 (1.6)
Laypersons*	0 (0)	2 (0.5)	2 (0.5)	5 (3.3)	9 (0.9)
Missing data**	5 (7.3)	6 (1.6)	0 (0)	2 (1.3)	13 (1.3)
Total	69 (100)	379 (100)	428 (100)	152 (100)	1028 (100)
**Origins of reports**					
North District	36 (52.2)	263 (69.4)	145 (33.9)	49 (32.2)	493 (48.0)
Central District	2 (2.9)	9 (2.4)	97 (22.7)	68 (44.8)	176 (17.1)
South District	22 (31.9)	54 (14.3)	45 (10.5)	15 (9.9)	136 (13.2)
Eastern District	9 (13.0)	47 (12.4)	117 (27.3)	12 (7.9)	185 (18.0)
Missing data	0 (0)	6 (1.6)	24 (5.6)	8 (5.3)	38 (3.7)
Total	69 (100)	379 (100)	428 (100)	152 (100)	1028 (100)

*TADRRS-HM* Taiwan Adverse Drug Reaction Reporting System for Herbal Medicine.

*Patients and caregivers.

**Missing data refer to ADR records that were incompletely filled.

^#^ADR records were filled by medical personnel, without attribution of the person’s professional status.

### Causality analysis

Table 3 displays the type of adverse reaction, causality and severity of ADR events. Most ADR events were not serious adverse events (SAES; n = 887; 86.7%); 136 (13.3%) were classified as SAEs (considered to be life-threatening or requiring inpatient hospitalization; none was fatal); 733 (71.3%) were caused by unexpected side effects; 221 (21.5%) were due to excessively enhanced drug effects.

**Table 3:** Basic information from 1028 ADR reports in the TADRRS-HM (1998–2016).

Basic information from 1028 ADR reports	Numbers of ADR reports n (%)
**Serious adverse events**	
Yes	136 (13.3)
No	887 (86.7)
Missing data*	5 (0.0)
**Type of adverse reactions**	
Excessively enhanced drug effects**	221 (21.5)
Unexpected side effects^#^	733 (71.3)
Missing data	74 (7.2)
**Severity (modified Hartwig and Siegel scale)**	
Mild	744 (72.4)
Moderate	179 (17.4)
Severe	67 (6.5)
Missing data	38 (3.7)
**Naranjo score**	
Doubtful (≤ 0)	135 (13.1)
Possible (1–4)	608 (59.1)
Probable (5–8)	267 (26.0)
Definite (≥ 9)	18 (1.8)

*TADRRS-HM* Taiwan Adverse Drug Reaction Reporting System for Herbal Medicine.

*Missing data refer to ADR records that were incompletely filled.

**Excessively enhanced drug effects refer to those that occur in patients prescribed a normal therapeutic dosage who experience amplified side effects from the medicine due to their individual characteristics. For example, during normal use/dosage, licorice can result in abdominal fullness, but some patients may experience excessive abdominal fullness.

^#^Unexpected side effects are those that are previously unknown in relation to a particular herb.

According to the Modified Hartwig and Siegel scale, the ADRs were classified as mild in the majority of cases (n = 744; 72.4%); far fewer were classified as moderate (n = 179; 17.4%), severe (n = 67; 6.5%), or could not be classified due to missing data (n = 38; 3.7%). Naranjo scores for causality in relation to all AEs were doubtful (score ≤ 0; n = 135; 13.1%) or possible (n = 608; 59.1%) in the majority of cases; fewer than one-third were scored as probable (n = 267; 26.0%), or definite (n = 18; 1.8%).

### System organ class

The most commonly reported SOCs in the 1028 ADR events are displayed in Table 4. Gastrointestinal system disorders (n = 353; 33.4%), such as abdominal pain, constipation, and diarrhea, were the most common ADRs followed by skin and subcutaneous tissue disorders (n = 224; 21.2%), such as pruritus and rash maculopapular. Immune system diseases (n = 158; 14.9%) consisted of allergic reactions, while nervous system disorders (n = 123; 11.6%) consisted of dizziness, numbness, and syncope. Other SOCs detailed in Table 4 occurred at frequencies of < 10%.

**Table 4:** Numbers of adverse events in the TADRRS-HM (1998–2016), grouped by System Organ Class.

System Organ Class	Numbers of ADR reports (n = 1028)
	n (%)
Gastrointestinal system disorders	353 (33.4)
Skin and subcutaneous tissue disorders	224 (21.2)
Immune system diseases	158 (14.9)
Nervous system disorders	123 (11.6)
General disorders	78 (7.4)
Cardiac disorders	75 (7.1)
Renal and urinary disorders	63 (6.0)
Respiratory, thoracic and mediastinal disorders	55 (5.2)
Psychiatric disorders	50 (4.7)
Hepatobiliary diseases	45 (4.3)
Investigations	45 (4.3)

*TADRRS-HM* Taiwan Adverse Drug Reaction Reporting System for Herbal Medicine.

### The most commonly reported herbal medicines, herbal formulas and folk herbals

Table 5 displays the most commonly reported single herbs, herbal formulas and folk herbals in the 893 cases analyzed from the TADRRS-HM. The top five ADR reports of single herbs involved Aconiti Radix (n = 22), Ephedrae Herba (n = 18), Glycyrrhizae Radix et Rhizoma (n = 17), Salviae Miltiorrhizae Radix et Rhizoma (n = 17), and Angelicae Sinensis Radix (n = 17). The top five herbal formulas in the ADR reports involved Xiao-Qing-Long-Tang (n = 21), Ma-Xing-Gan-Shi-Tang (n = 20), Jia-Wei-Xiao-Yao-San (n = 20), Xie-Fu-Zhu-Yu-Tang (n = 18), and Zhi-Bo-Di-Huang-Wan (n = 17). The top five folk herbals involved *Datura suaveolens* (n = 11), *Alocasia macrorrhiza* (n = 4), *Typhonium divaricatum* (n = 4), *Taxus chinensis* (n = 3), and *Dioscorea bulbifera* (n = 3). The Supplementary files contain details of the suspected AEs of Aconiti Radix, Ephredrae Herba, Xiao-Qing-Long-Tang, and *Datura suaveolens*.

**Table 5:** The top 10 frequently reported herbals and formulas in TADRRS-HM, 1998–2016.

Rank*	Single herbs (n = 2733)	*n*	Rank	Herbal formulas (n = 2909)	*n*	Rank	Folk herbals (n = 558)	*n*
1	Aconiti Radix	22	1	Xiao-Qing-Long-Tang	21	1	*Datura suaveolens*	11
2	Ephedrae Herba	18	2	Ma-Xing-Gan-Shi-Tang	20	2	*Alocasia macrorrhiza*	4
3	Glycyrrhizae Radix et Rhizoma	17	2	Jia-Wei-Xiao-Yao-San	20	2	*Typhonium divaricatum*	4
3	Salviae Miltiorrhizae Radix et Rhizoma	17	4	Xie-Fu-Zhu-Yu-Tang	18	4	*Taxus chinensis*	3
3	Angelicae Sinensis Radix	17	5	Zhi-Bo-Di-Huang-Wan	17	4	*Dioscorea bulbifera*	3
6	Rhei Radix ET Rhizoma	16	6	Ban-Xia-Xie-Xin-Tang	16			
7	Zingiberis Rhizoma	13	7	Liu-Wei-Di-Huang-Wan	13			
8	Rehmanniae Radix	11	7	Xiang-Sha-Liu-Jun-Zi-Tang	13			
8	Coptidis Rhizoma	11	9	Tian-Wang-Bu-Xin-Dan	12			
10	Xanthii Fructus	10	10	Ge-Gen-Tang	11			
10	Cicadae Periostracum	10	10	Long-Dan-Xie-Gan-Tang	11			

*TADRRS-HM* Taiwan Adverse Drug Reaction Reporting System for Herbal Medicine.

*Frequency of reports involving suspected herbal substances with a Naranjo scale score of ≥ 1 (a score of 1–4 is considered "possible", 5–8 "probable", and ≥ 9 "definite", as to the likelihood of the herb causing the ADR)^24^.

### ADR manifestations of Aconiti Radix

Table 6 details the suspected AEs in ADRs relating to Aconiti Radix, the most frequently reported single herb. Most of the suspected AEs were classified under SOC nervous system disorders (n = 13), cardiac disorders (n = 10), gastrointestinal system disorders (n = 8), and general disorders and administration site conditions (n = 8). The suspected AEs in ADR reports relating to the most frequently reported herbal formula (Xiao-Qing-Long-Tang) and folk herbal (*Datura suaveolens*), as well as the second most frequently reported single herb Ephedrae Herba, are detailed in the Supplementary materials (Tables S1-3).

**Table 6:** The suspected AEs in ADRs relating to Aconiti Radix, the most frequently reported single herb (22 ADR reports).

System organ class	Adverse event	n
Gastrointestinal system disorders	Nausea	1
	Dry mouth	4
	Diarrhea	2
	Constipation	1
Infections and infestations	Allergic reaction	2
Nervous system disorders	Paresthesia	6
	Dizziness	6
	Syncope	1
Psychiatric disorders	Delirium	1
	Insomnia	4
Respiratory, thoracic and mediastinal disorders	Dyspnea	1
	Laryngeal inflammation	1
Musculoskeletal and connective tissue disorders	Flank pain	2
Skin and subcutaneous tissue disorder	Pruritus	1
	Rash acneiform	2
General disorders and administration site conditions	Fever	1
	Malaise	2
	Edema limbs	2
	Edema face	1
	Localized edema	2
Cardiac disorders	Palpitations	3
	Sinus bradycardia	4
	Ventricular arrhythmia	1
	Chest pain—cardiac	2
Kidney and urinary system diseases	Acute kidney injury	1
	Dysuria	3
Vascular disorders	Hypotension	4

### The SAE cases

Of the 136 SAE cases, 78 presented with severe clinical manifestations and had Naranjo scores of 1 or greater (a score of 1–4 is considered “possible”, 5–8 “probable”, and ≥ 9 “definite”, as to the likelihood of the herb causing the ADR). Among these 78 SAE cases, 21 were associated with “unknown” herbal medicines or herbal formulas, while another 18 cases were associated with folk herbs such as *Datura suaveolens*, *Breynia officinalis*, *Abrus precatorius*, etc. Only 7 cases reported TCM formulas such as Si-Ni-Tang, Fang-Feng-Tong-Sheng-San, Ma-Xing-Gan-Shi-Tang, Long-Dan-Xie-Gan-Tang, etc.; the first formula contains Ephredra Herba, the second and third formulas contain Aconiti Radix.

Eight deaths were associated with SAEs. The suspected herbals causing death were reported to be Cinnabar, *Fructus Xanthii*, Ephedrae Herba, *Cassiae Occidentalis* Semen, *Bufonis Venenum* (Toad Venom), Realgar and unknown drugs. We identified 3 cases involving arsenic; 2 involving Realgar (arsenic sulfide) and 1 involving arsenic trioxide (Asadin®), with side effects including nausea, vomiting, diarrhea, and also darkening of skin on the body, similar to that seen with Blackfoot disease, as well as 1 death due to poisoning from cinnabar (mercury sulfide) incense vapor.

In our dataset of SAE cases, 4 involved *Averrhoa carambola* (starfruit), which has been listed in the *Compendium of Materia Medica* since the sixteenth century; in all 4 cases, patients had chronic renal insufficiency and developed acute renal failure after ingesting starfruit.

## Discussion

This analysis of ADRs reported in Taiwan from 1998 to 2016 revealed 1028 ADRs involving single herbs, herbal formulas and folk herbals, most of which were classified by severity as mild or moderate in intensity, and around one-third were classified as gastrointestinal system disorders. Moreover, most ADRs involved adults aged 35–65 years, which contradicts the notion that children and the elderly are more vulnerable to developing ADRs. Our finding might be because adults are more likely to use herbal medicine treatments and to be concerned about protecting their health status. It may also be that this age group (35–65 years) has greater health literacy than elderly people (and certainly more so than children). The finding that most patients in the ADR reports were females may reflect the fact that more females than males use TCM in Taiwan^26^, rather than the supposition that females are more sensitive than males to ADRs caused by herbal medicines.

We used the Naranjo Adverse Drug Reactions Probability Scale, a widely used causality assessment tool that provides an objective, standardized approach for assessing ADR causality in relation to an administered drug or herb^24,27,28^. The Naranjo scale scored fewer than one-third of AEs in the ADR reports as "probably" (26.0%) or "definitely" (1.8%) caused by single herbs, herbal formulas, or folk herbals; the majority were "doubtful" (13.1%) or "possible" (59.1%), indicating that many of those events may be attributed to other causes, such as herbal-herbal interactions, herbal-drug interactions, food-herbal interactions, or environmental factors (e.g., pesticides, heavy metals, and molds) (Table 3). One criticism of the Naranjo Scale is that as it assesses general drug reactions, it cannot include critical elements related to the likelihood of drug-induced liver injury, such as specific time to onset, criteria for time of recovery, and list of critical diagnoses to exclude, making this scale inappropriate for attributing causality in hepatotoxicity^29^. These considerations led to the development of the RUCAM (Roussel Uclaf Causality Assessment Method) tool, which can quantitatively grade causality for individual suspect drugs/herbs in a hepatotoxicity case report and has been successfully applied by clinicians and practitioners caring for patients with suspected drug-induced or herb-induced liver injury^29^.

Our selection of the Naranjo Scale instead of the RUCAM for ADR causality was because our ADR reporting system does not record details associated with liver injury such as to time to onset of liver injury from the start of the drug/herb, risk factors for liver abnormalities (e.g., alcohol consumption, other classes of medications that are known to cause liver damage, illicit drug and substance abuse), results from liver function testing after cessation of the drug/herb, time taken for recovery, response to unintentional re-exposure of the drug/herb alone, or previous hepatotoxicity of the drug/herb. Moreover, the majority of ADR events reported in this data analysis were categorized under SOC gastrointestinal system disorders (33.4%); only 4.3% were assigned to SOC hepatobiliary diseases (Table 4), making it impractical to use an ADR causality tool focusing on liver injuries.

At the time of filing to TADRRS-HM, the minority of AEs in the ADR reports were classified as serious (13.3%) and the overwhelming majority were not (86.7%); SAE data were missing for 5 cases (Table 3). As for types of adverse reactions, most (71.3%) were classified as unexpected, previously unreported side effects and 21.5% were classified as excessively enhanced effects associated with the usual prescribed dosage (Table 3). As the ADR reports contained no information about dosage, it was impossible to determine whether any AEs were associated with overdoses. Analysis of the ADR reports for severity according to the Modified Hartwig and Siegel Scale scored most as mild (72.4%) or moderate (17.4%) in severity and 6.5% as severe; data were missing for 3.7% of the ADR reports and therefore could not be scored for severity.

As shown in Table 5, a total of 2733 instances of single herbs were listed amongst the 1028 ADR reports; the highest number (n = 22; 0.80%) involved Aconiti Radix, followed by Ephedrae Herba (n = 18; 0.66%), 17 (0.62%) involved Glycyrrhizae Radix et Rhizoma, 17 (0.62%) involved Salviae Miltiorrhizae Radix et Rhizoma, and 17 (0.62%) involved Angelicae Sinensis Radix. Most of the frequently reported single herbals in TADRRS-HM are widely used in many herbal formulas, which increases their total numbers of ADR reports. Of the 2909 instances of herbal formulas implicated in suspected AEs, four were ranked 1, 2 or 4: Rank 1, Xiao-Qing-Long-Tang (n = 21; 0.72%); Rank 2, Ma-Xing-Gan-Shi-Tang (n = 20; 0.69%); Rank 2, Jia-Wei-Xiao-Yao-San (n = 20; 0.69%); and Xie-Fu-Zhu-Yu-Tang (n = 18; 0.60%). Of the 558 instances of folk herbals mentioned, five were ranked 1, 2, or 4: Rank 1, *Datura suaveolens* (n = 11; 2.13%); Rank 2, *Alocasia macrorrhiza* (n = 4; 0.78%); Rank 2, *Typhonium divaricatum* (n = 4; 0.78%); Rank 4, *Taxus chinensis* (n = 3; 0.50%); and Rank 4, *Dioscorea bulbifera* (n = 3; 0.50%).

Processing techniques may alter the properties of herbal medicines; in some cases, processing increases or alters the therapeutic effect, while in other cases it lowers the toxicity or reduces undesirable adverse effects. Improper processing of herbal medicines can lead to adverse reactions. For example, Aconiti Radix is derived from the lateral root of *Aconitum carmichaeli Debx*., a medicinal herb that is commonly used in TCM prescriptions for cardiovascular diseases, rheumatoid arthritis, and bronchitis. In its raw form (mostly for external use), Aconiti Radix is highly toxic. After appropriate processing, the toxicity is reduced and Aconiti Radix may be used for oral administration, or it can be cooked with dried ginger and licorice to reduce toxicity. The principal bioactive ingredients of Aconiti Radix (aconitine, mesaconitine, and hypoconitine) are alkaloid toxins that are potentially cardiotoxic and neurotoxic^30,31^. When processed appropriately, aconitine can be transformed by hydrolysis reaction into benzoylaconine or aconine, which are less toxic than aconitine^30,31^. Similarly, the toxicity of mesaconitine and hypoconitine can be lowered through hydrolysis reaction and appropriate processing into much less poisonous mesaconine and hypoconine. Aconitine is often used in herbal medicines for its cardiotonic, antirheumatic, anti-inflammatory and analgesic properties^31^. Improper processing and use of herbal decoctions containing aconitine have led to severe poisoning and even death, according to reports from China and other parts of Asia^31^.

Clinical management of aconite toxicity usually involves cardiovascular supportive treatment, as there is no specific therapy^32–35^. Clinical toxicity of aconite varies depending on the dosage used and the patient's clinical condition (frailty); no specific dose–response relationships have been identified in the clinical studies in the literature^35^. In our analysis of suspected AEs relating to Aconiti Radix, most were classified under SOC nervous system disorders (n = 13), cardiac disorders (n = 10), gastro-intestinal system disorders (n = 8), and general disorders and administration site conditions (n = 8) (Table 6). Less frequently reported AEs were categorized under SOC psychiatric disorders (n = 5), kidney and urinary system diseases (n = 4), vascular disorders (n = 4), skin and subcutaneous tissue disorders (n = 4), respiratory, thoracic and mediastinal disorders (n = 2), and musculoskeletal and connective tissue disorders (n = 2).

Ephedrae Herba has traditionally been prescribed in Chinese medicine for the treatment of asthma, fever and rhinorrhea^36^, and more recently as a dietary supplement for short-term weight loss^37–39^. However, although the pharmacologically active alkaloid of Ephedra, ephedrine, causes weight loss by reducing appetite and enhancing lipid metabolism, it is associated with significant AEs, which has led to the ban of ephedrine-containing supplements in the USA^39^. Those AEs include cardiovascular dysfunction, gastro-intestinal irritability, and CNS stimulation^36,39,40^.

Reports involving large doses of Ephedrae Herba products describe symptoms of nervousness, headaches, insomnia, dizziness, palpitations, skin flushing and tingling, and vomiting^41^. Investigations into the effects of Ephedrae Herba on cellular and humoral immune responses in mice have identified stimulation of the central nervous system, nausea, tremors, tachycardia, and urine retention as the principal AEs^42^. Our analysis of the ADR reports identified 18 suspected AEs (0.66%) involving Ephedrae Herba, most of which were classified under SOC nervous system disorders (n = 8) and cardiac disorders (n = 8). Other SOC classifications included gastrointestinal system disorders (n = 5), psychiatric disorders (n = 4), general disorders and administration site conditions (n = 3), skin and subcutaneous tissue disorders (n = 3), infections and infestations (n = 2), and 1 AE each in SOC kidney and urinary system diseases, vascular disorders, investigations, metabolism and nutrition disorders. A detailed breakdown of these cases is provided in the Supplementary files (Table S1).

Glycyrrhizae Radix et Rhizoma (licorice) is a commonly prescribed traditional herbal medicine used in many herbal medical formulas. Its function in TCM is to fortify the spleen, dispel phlegm to suppress cough and moisten the lung, relax tension and relieve pain, clear heat and detoxify, and harmonize the nature of medical formulas to reduce their strengths or side effects. It is extensively used in TCM to treat hepatitis, influenza, cough, and gastric ulcers^43^. In our analysis of ADR data involving Glycyrrhizae Radix et Rhizoma, suspected AEs mainly involved the kidneys, characterized by edema and hypokalemia, as well as allergic reactions of the immune system (urticaria). Patients with chronic diseases must pay particular attention to dosage, as these patients often use herbal medicine on a long-term basis. Gradual accumulation of the medicine in the body can cause toxic effects.

In our analysis of ADR reports, 21 cases involved the herbal formula Xiao-Qing-Long-Tang, with suspected AEs that were categorized under SOC gastrointestinal system disorders (n = 7), general disorders and administration site conditions (n = 5), kidney and urinary system diseases (n = 5), psychiatric disorders (n = 4), skin and subcutaneous tissue disorders (n = 4), cardiac disorders (n = 3), infections and infestations (n = 2), nervous system disorders (n = 2), musculoskeletal and connective tissue disorders (n = 2), respiratory, thoracic and mediastinal disorders (n = 1), eye disorders (n = 1), and reproductive system and breast disorders (n = 1). Details of the suspected AEs are provided in the Supplementary files (Table S2). Xiao-Qing-Long-Tang, also known as So-Cheong-Ryong-Tang or Sho-seiryo-to, has been used to treat patients with allergic rhinitis, bronchitis, bronchial asthma in Oriental countries for several centuries^44^.

Among the herbal formulas listed in Table 5, most cases are derived from the Solanaceae or Araceae families, containing plants with some compounds that can be toxic; these plants are commonly used in folk remedies. The toxic properties of *Datura suaveolens* are due to ingestion of its parasympatholytic alkaloids; systemic effects of intoxication with *Datura suaveolens* include several peripheral and central effects such as excitement and confusion, visual and auditory hallucinations, tachycardia, dry mouth, and dry flushed skin^45^. The 11 suspected AEs associated with *Datura suaveolens* in the ADR reports were mostly classified under SOC nervous system disorders (n = 17), followed by gastrointestinal system disorders (n = 8), psychiatric disorders (n = 8) and vascular disorders (n = 5) (Supplementary Table S3). All of the suspected AEs are detailed in the Supplementary files. Accidental consumption of *Datura suaveolens* may cause delirium, hallucination, and tachycardia, etc.

Currently, most reports of adverse reactions to herbal medicines have emanated from China or other countries^7–11^. Based on reports detailing ADR cases originating from China, several main causes for adverse reactions to herbal medicine are identified, including the following: confusion surrounding the many types of herbal medicines; improper processing techniques; incorrect dosages; harmful effects arising from interactions between different herbs; a mismatch between the prescribed formula and actual disease pattern, abuse and misuse of folk remedies; incorrect decoction methods; and adverse reactions caused by individual factors such as the particular characteristics of an individual’s body type or immune system. Ingesting an inappropriate quantity of herbal medicines can also produce adverse effects. Owing to the popular perception of the non-toxic nature of herbal medicines, consumers often take large doses to hasten recovery. In the TADRRS-HM, we have found three ADR cases related to the medicinal herb Chuan-Xiong, the rhizome of *Ligusticum chuanxiong* Hort. Normally, Chuan-Xiong promotes blood circulation and dispels blood stasis^46^. A small dose of Chuan-Xiong stimulates the myocardium and causes uterine contractions; a large dose inhibits the myocardium and induces vasodilation, which may cause a fatal decrease in blood pressure.

In the TADRRS-HM, we identified 6 ADR cases related to ginseng root (*Panax ginseng*), including allergic reactions such as flushing, dry eyes, and drug eruption, and and heart palpitations. Owing to the prevalent warming and tonic culture, ginseng root is often used for medical treatment or as a dietary supplement^47^. Some adverse reactions may be attributable to individual factors such as the particular characteristics of an individual’s body type or the immune system. Furthermore, herbal medicines are often added to many proprietary food items as taste enhancers or as a marketing tool. Ginseng essence, ginseng oral solution, ginseng medicinal liquor, and other health foods have flooded the market, which can also easily lead to related adverse reactions. In certain cases, harmful effects arise from interactions between different herbs. Some hazardous interactions between specific herbs are so well documented that the concomitant use of those drugs is forbidden. For example, ginseng root should not be combined with the vegetable product daikon; in herbal medicine terminology, ginseng “fears” daikon.

There are 7 ADR cases associated with Si-Wu-Tang in the TADRRS-HM. Marketing campaigns and the prevalent concept of tonication in the Chinese society have propelled the evolution of Si-Wu-Tang from a traditional trauma medicine for invigorating blood circulation into a health-preserving drink targeted at women. The widespread over-the-counter availability of herbal medicines has increased the incidence of adverse reactions. The inappropriate use of cinnamon-containing foods is an excellent example of this phenomenon. Patients with a common cold often use cinnamon and lozenges, which aggravate respiratory tract inflammation. When used for flavoring food, cinnamon is typically used in small doses. When used in a disease state, cinnamon may cause adverse reactions. Notably, the risk of ADR events is higher with TCM injections compared with all other TCM dosage formulations, and with a much higher number of ADRs compared with conventional injections^48^.

Many adverse reactions recorded in the TADRRS-HM database were attributable to medicinal materials taken in the form of drugs or food, and the relevant information and severity was documented in the database. This database can provide empirical data for management of many controversial herbal medicinal materials with homology of medicine and food; this empirical data could facilitate the formulation of relevant laws and regulations to control the use of these medicinal items.

### Study limitations

In this analysis, ADRs of herbal medicines related to many aspects of their usage, including inappropriate dosages, route of administration, and different disease patterns. Some of the ADR cases were difficult to assess, because the ADR reports lacked complete information (e.g., follow-up details were missing, or the TCM-related records did not include the pattern diagnosis). As some patients used herbal medicine concurrently with Western medicine, or combined several single herbals with more than one herbal formula, it was difficult to attribute certain ADRs to the single herbals or herbal formulas, because the ADR reports did not include detailed information about the concurrent use of herbal medicines or Western medicines. Lastly, as the vast majority of the reporting is generated by health professionals, spontaneous reporting systems generally omit information from patients. Thus, it is likely that the true incidence of ADR events is under-reported in Taiwan.

## Conclusions

The TADRRS-HM contains data for 1028 cases reported from 1998 to 2016. We believe that this system will confer practical, long-term benefits for the epidemiological analysis of adverse reactions to herbal medicines in Taiwan. The ADR reports generated by the TADRRS-HM can help to characterize the potential ADRs of individual herbs and formulas. Moreover, the data indicate that herbal medicines may cause a wide range of ADRs and that most SAEs occur in the absence of medical professionals. How to modernize, socialize, and integrate the knowledge and skills associated with traditional herbal medicine with those of the current health care system is an issue that deserves close attention, to ensure the safe, most effective therapeutic use of these medicines.

## Supplementary Information

Supplementary Information.

## Abbreviations

ADR: Adverse drug reaction
AE: Adverse event
TCM: Traditional herbal medicine
TADRRS-HM: Taiwan Adverse Drug Reaction Reporting System for Herbal Medicine
