# Supplementary material for: A system for reporting and evaluating adverse drug reactions of herbal medicine in Taiwan from 1998 to 2016
Source: Sci Rep. 2021 Nov 2;11:21476. doi: 10.1038/s41598-021-00704-w (PMC8564513; doi:10.1038/s41598-021-00704-w)
Supplement: Supplementary file 1 — Supplementary Information. [file 41598_2021_704_MOESM1_ESM.docx]

**Supplementary materials**

Table S1. The suspected AEs in ADRs relating to Ephedrae Herba, the second most frequently reported folk herbal (18 ADR reports)

| System Organ Class | Adverse Event | n |
| --- | --- | --- |
| Gastro-intestinal system disorders | Nausea | 1 |
|  | Dry mouth | 1 |
|  | Diarrhea | 1 |
|  | Vomiting | 1 |
|  | Abdominal pain | 1 |
| Nervous system disorders | Depressed level of consciousness | 1 |
|  | Dizziness | 2 |
|  | Syncope |  |
|  | Stroke | 2 |
|  | Intracranial hemorrhage | 1 |
|  | Headache | 1 |
|  | Other | 1 |
| Infections and infestations | Allergic reaction | 2 |
| Psychiatric disorders | Insomnia | 4 |
| Skin and subcutaneous tissue disorder | Pruritus | 2 |
|  | Hyperhidrosis | 1 |
| General disorders and administration site conditions | Malaise | 3 |
| Cardiac disorders | Palpitations | 7 |
|  | Ventricular arrhythmia | 1 |
| Kidney and urinary system diseases | Dysuria | 1 |
| Vascular disorders | Hypertension | 1 |
| Investigations | Weight loss | 1 |
| Metabolism and nutrition disorders | Anorexia | 1 |
|  |  |  |

Table S2. The suspected AEs in ADRs relating to Xiao-Qing-Long-Tang, the most frequently reported herbal formula (26 ADR reports)

| System Organ Class | Adverse Event | n |
| --- | --- | --- |
| Gastro-intestinal system disorders | Vomiting | 1 |
|  | Dry mouth | 4 |
|  | Diarrhea | 1 |
|  | Other | 1 |
| Infections and infestations | Allergic reaction | 2 |
| Nervous system disorders | Dizziness | 2 |
| Psychiatric disorders | Insomnia | 4 |
| Respiratory, thoracic and mediastinal disorders | Epistaxis | 1 |
| Musculoskeletal and connective tissue disorders | Flank pain | 2 |
| Skin and subcutaneous tissue disorder | Alopecia | 1 |
|  | Pruritus | 3 |
| General disorders and administration site conditions | Fever | 3 |
|  | Malaise | 1 |
|  | Edema limbs | 1 |
| Cardiac disorders | Palpitations | 2 |
|  | Chest pain - cardiac | 1 |
| Kidney and urinary system diseases | Dysuria | 5 |
| Eye disorders | Other | 1 |
| Reproductive system and breast disorders | Vaginal discharge | 1 |
| Infections and infestations | Folliculitis | 1 |
|  |  |  |

Table S3. The suspected AEs in ADRs relating to *Datura suaveolens*, the most frequently reported folk herbal (11 ADR reports)

| System Organ Class | Adverse Event | n |
| --- | --- | --- |
| Gastro-intestinal system disorders | Nausea | 2 |
|  | Vomiting | 4 |
|  | Dry mouth | 2 |
| Nervous system disorders | Depressed level of consciousness | 4 |
|  | Dizziness | 6 |
|  | Syncope | 3 |
|  | Memory impairment | 1 |
|  | Spasticity | 1 |
| Psychiatric disorders | Hallucinations | 3 |
|  | Delirium | 2 |
|  | Confusion | 3 |
| Respiratory, thoracic and mediastinal disorders | Dyspnea | 1 |
| Eye disorders | Blurred vision | 2 |
| Musculoskeletal and connective tissue disorders | Muscle cramp | 1 |
| Vascular disorders | Flushing | 3 |
|  | Hot flashes | 2 |
| Cardiac disorders | Supraventricular tachycardia | 1 |
| Kidney and urinary system diseases | Dysuria | 2 |
| General disorders and administration site conditions | Malaise | 2 |
|  |  |  |
